# The impact of Ti and temperature on the stability of Nb_5_Si_3_ phases: a first-principles study

**DOI:** 10.1080/14686996.2017.1341802

**Published:** 2017-07-10

**Authors:** Ioannis Papadimitriou, Claire Utton, Panos Tsakiropoulos

**Affiliations:** ^a^ Department of Materials Science and Engineering, The University of Shefﬁeld, Shefﬁeld, UK

**Keywords:** Ab initio calculations, phase transitions, elastic constants, enthalpy of formation, coefficient of thermal expansion, intermetallic phases, 10 Engineering and Structural materials, 401 1st principle calculations, 106 Metallic materials / Refractory metal intermetallic alloys / Nb silicide based alloys

## Abstract

Nb-silicide based alloys could be used at T > 1423 K in future aero-engines. Titanium is an important additive to these new alloys where it improves oxidation, fracture toughness and reduces density. The microstructures of the new alloys consist of an Nb solid solution, and silicides and other intermetallics can be present. Three Nb_5_Si_3_ polymorphs are known, namely αNb_5_Si_3_ (*tI*32 Cr_5_B_3_-type, D8_l_), βNb_5_Si_3_ (*tI*32 W_5_Si_3_-type, D8_m_) and γNb_5_Si_3_ (*hP*16 Mn_5_Si_3_-type, D8_8_). In these 5–3 silicides Nb atoms can be substituted by Ti atoms. The type of stable Nb_5_Si_3_ depends on temperature and concentration of Ti addition and is important for the stability and properties of the alloys. The effect of increasing concentration of Ti on the transition temperature between the polymorphs has not been studied. In this work first-principles calculations were used to predict the stability and physical properties of the various Nb_5_Si_3_ silicides alloyed with Ti. Temperature-dependent enthalpies of formation were computed, and the transition temperature between the low (α) and high (β) temperature polymorphs of Nb_5_Si_3_ was found to decrease significantly with increasing Ti content. The γNb_5_Si_3_ was found to be stable only at high Ti concentrations, above approximately 50 at. % Ti. Calculation of physical properties and the Cauchy pressures, Pugh’s index of ductility and Poisson ratio showed that as the Ti content increased, the bulk moduli of all silicides decreased, while the shear and elastic moduli and the Debye temperature increased for the αNb_5_Si_3_ and γNb_5_Si_3_ and decreased for βNb_5_Si_3_. With the addition of Ti the αNb_5_Si_3_ and γNb_5_Si_3_ became less ductile, whereas the βNb_5_Si_3_ became more ductile. When Ti was added in the αNb_5_Si_3_ and βNb_5_Si_3_ the linear thermal expansion coefficients of the silicides decreased, but the anisotropy of coefficient of thermal expansion did not change significantly.

## Graphical abstract

**Figure:**
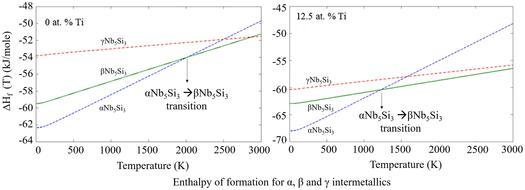

## 1. Introduction

The development of high-temperature engineering alloys that can operate at temperatures above those of the latest generations of Ni-based superalloys is a priority in current metallurgical research to enable future gas turbine technologies to meet environmental and performance targets [1]. The Nb-silicide based alloys have higher melting temperatures, lower densities and better creep properties and are stable at higher temperatures than the Ni-based superalloys. These new alloys are also known as Nb *in situ* composites, and their microstructures consist of Nb solid solution that provides toughness and intermetallics that give low- and high-temperature strength and creep resistance [2]. Different alloying additions are used to achieve a balance of properties, in particular room-temperature fracture toughness, low- and high-temperature oxidation resistance and strength and creep [1,2].

The Nb_5_Si_3_ is the desirable intermetallic in these new alloys. Three polymorphs of Nb_5_Si_3_ are reported, namely the αNb_5_Si_3_ (*tI*32 Cr_5_B_3_-type, D8_l_), βNb_5_Si_3_ (*tI*32 W_5_Si_3_-type, D8_m_) and γNb_5_Si_3_ (*hP*16 Mn_5_Si_3_-type, D8_8_). The αNb_5_Si_3_ and βNb_5_Si_3_ are the 5–3 silicides in the binary equilibrium Nb-Si phase diagram [3], both have tetragonal crystal structure, which contains 20 atoms of Nb and 12 atoms of Si, but crystallize in different atomic arrangements. The γNb_5_Si_3_ silicide is hexagonal with 10 Nb atoms and 6 Si atoms and is considered metastable [3]. In the Nb-Si binary phase diagram αNb_5_Si_3_ transforms to βNb_5_Si_3_ at 2208 K [3].

The addition of Ti in Nb-silicide based alloys not only reduces their density but also improves their fracture toughness and oxidation resistance [2,4,5]. To achieve a balance of properties, the concentration of Ti in Nb-silicide based alloys must be optimized because Ti (i) does not increase the ductile to brittle transition temperature (DBTT) of bcc Nb for concentrations up to ≈ 24 at. %, (ii) has the weakest effect of all additions X on the yield strength at T = 1095 °C and high-temperature strength at T = 1200 °C of Nb-X solid solution alloys, where X is transition (including refractory) metal [6] and (iii) substitutes for Nb in (Nb,Ti)_5_Si_3_ silicides and increases the toughness of unalloyed Nb_5_Si_3_ from about 3 MPa√m to about 10 MPa√m at Ti ≈ 25 at. %, but at higher Ti contents the hexagonal (Ti,Nb)_5_Si_3_ is stabilized and the toughness drops to values below 3 MPa√m [4]. The stable structure for the fully Ti-substituted end member, i.e. the Ti_5_Si_3_, is hexagonal (*hP*16 Mn_5_Si_3_-type, D8_8_). The Ti_5_Si_3_ is isomorphous with γNb_5_Si_3_.

Even though Ti is an important addition, there is lack of data in the literature about the effect that Ti has on the stability of the different Nb_5_Si_3_ polymorphs. The effect of alloying with Ti on the transformation temperature between the two tetragonal polymorphs has not been reported, nor has the effect of Ti on their coefficient of thermal expansion (CTE). However, it has been shown that high concentrations of Ti in (Ti,Nb)_5_Si_3_ stabilized the 5–3 silicide in the hexagonal crystal structure in Nb-silicide based alloys at temperatures below 1500 °C [7,8]. The latter is undesirable because the hexagonal 5–3 silicide is reported to have inferior creep properties than the αNb_5_Si_3_ and βNb_5_Si_3_ [1,2]. The CTE of Ti_5_Si_3_ is also significantly more anisotropic [9].

The early data that were used to construct liquidus projection of the Nb-Ti–Si ternary system did not identify which was the structure of 5–3 compound(s) in the cast alloys (i.e. authors did not clarify which 5–3 polymorph was formed), and the projection gave a primary Nb_5_Si_3_ solidification area without specifying whether the primary silicide was the βNb_5_Si_3_ or the αNb_5_Si_3_ or the hexagonal Ti_5_Si_3_ based 5–3 silicide [10]. Geng et al. [11] proposed a liquidus projection for the Nb-Ti–Si ternary system with a large primary αNb_5_Si_3_ solidification area. Li et al. [12] revised the Nb-Ti–Si liquidus projection based on a study of ternary alloys in the Nb_5_Si_3_-Ti_5_Si_3_ region. The proposed liquidus projection by Li et al. shows that primary βNb_5_Si_3_ will form for Ti concentrations up to approximately 40 at. %, the liquidus projection has a very narrow primary αNb_5_Si_3_ solidification area and indicates that at higher concentrations primary hexagonal Ti_5_Si_3_ will form during solidification. A similar liquidus projection was proposed recently by Jânio Gigolotti et al. [13], with an extended βNb_5_Si_3_ region and narrow αNb_5_Si_3_ area. No primary αNb_5_Si_3_ solidification area is shown in the Nb-Ti–Si liquidus projection by Bulanova and Fartushna [14]. There are no data about the transformation temperature of 5–3 silicides alloyed with Ti below the liquidus.

In this work first-principles calculations are used to study the stability and physical properties of the three polymorphs, αNb_5_Si_3_, βNb_5_Si_3_ and γNb_5_Si_3_ alloyed with Ti (up to 12.5 at. % Ti for αNb_5_Si_3_ and βNb_5_Si_3_ and up to 50 at. % Ti for γNb_5_Si_3_). Density functional theory (DFT) is used to study the enthalpy of formation and properties of the αNb_5_Si_3_, βNb_5_Si_3_ and γNb_5_Si_3_ compounds with and without Ti additions at T = 0 K. To probe the effect of Ti on the transformation temperatures, the temperature dependence of the heats of formation of the compounds is computed by incorporating phonon calculations. The paper provides new data that advance current understanding of the stability of complex Nb-silicide based alloys and the design and development of new alloys.

## 2. Computational details

The CASTEP (Cambridge Serial Total Energy Package) code [15] was used for the calculations, as described by Papadimitriou et al. [16]. The valences for the atomic configurations were Nb-4s^2^4p^6^4d^4^5s^1^, Ti-3s^2^3p^6^3d^2^4s^2^ and Si-3s^2^3p^2^. An energy cut-off of 500 eV was sufficient to reduce the error in the total energy to less than 1 meV/atom. A Monkhorst–Pack k-point grid separation of 0.03 Å^−1^ was used for the integration over the Brillouin zone according to the Monkhorst–Pack scheme [17]. Geometry optimizations of the structures were performed with thresholds for converged structures less than 1 × 10^−7^ eV, 1 × 10^−3^ eV/Å, 1 × 10^−4^ Å and 0.001 GPa, respectively, for energy change per atom, maximum residual force, maximum atomic displacement and maximum stress.

The method of finite displacements was used [16]. The forces on atoms were calculated when slightly perturbing the ionic positions [18]. The supercells used were as follows: 4 × 4 × 4 for Nb, 4 × 4 × 3 for Ti, 3 × 3 × 3 for Si, 2 × 2 × 2 for γNb_5_Si_3_ and Ti_5_Si_3_ and 2 × 2 × 1 for αNb_5_Si_3_ and βNb_5_Si_3_. The vibrational contributions to the enthalpy, entropy, free energy and heat capacity versus temperature and the Debye temperature were obtained using the quasiharmonic approximation [16]. The phonon density of states (DOS) of each element separately was calculated to obtain the finite temperature enthalpy of formation.

The linear thermal expansion coefficients (α) were obtained by generating structures with increasing the ratios a/a_0_ and c/c_0_ (a_0_ and c_0_ are the lattice parameters in the ground state) from 0.991 to 1.006 with an increment of 0.003 and conducting a phonon calculation for each volume. The equilibrium lattice parameters a(T,P) and c(T,P) were then calculated at every given temperature using the quasi-harmonic approximation by minimizing the total free energy with respect to volume, thus finding the equilibrium volume at each temperature. After calculating the a(T,P) and c(T,P) the linear thermal expansion coefficients α_a_ and α_c_ were obtained. This procedure was repeated for αNb_5_Si_3_, βNb_5_Si_3_, αNb_16_Ti_4_Si_12_ and βNb_16_Ti_4_Si_12_.

The elastic constants and properties were calculated as described in Papadimitriou et al. [16]. The calculation method consisted of applying a given strain and calculating the stress. At each deformation the unit cell was kept fixed, and the internal coordinates were optimized. The matrix of the linear elastic constants was reduced according to the crystal structure of each phase. The maximum number of strain patterns (sets of distortions) for a tetragonal or hexagonal structure is two and one for cubic cells. Six strain steps (varying from –0.003 to 0.003) were used [16].

For the cubic (Nb) and diamond (Si) structures a series of six geometry optimizations were done to evaluate the three independent elastic constants C_11_, C_12_ and C_44_, whereas for the tetragonal αNb_5_Si_3_ and βNb_5_Si_3_ and hexagonal Ti, γNb_5_Si_3_ and Ti_5_Si_3_ structures the corresponding number was twelve, with the six independent elastic constants being C_11_, C_12_, C_13_, C_33_, C_44_ and C_66_ for the tetragonal and C_11_, C_12_, C_13_, C_33_ and C_44_ for the hexagonal. After acquiring the matrix of the elastic constants and confirming that the mechanical stability criteria [19] are satisfied, the bulk (B), Young’s (E) and shear (G) moduli, Poisson’s ratio (ν) and Debye temperature were obtained as described in Papadimitriou et al. [16].

## 3. Results and discussion

### 3.1. Site occupancies, lattice constants and densities of states

Twelve structures in total were investigated in the current study, four for each of the αNb_5_Si_3_, βNb_5_Si_3_ and γNb_5_Si_3_ silicides. In all cases, each of the four structures contained an increasing number of Ti atoms, starting from 1 and increasing to 4. Thus, from the structure with the lowest Ti content to that with the highest, the corresponding percentages were 3.125, 6.25, 9.375 and 12.5 at. % Ti for the αNb_5_Si_3_ and βNb_5_Si_3_ and 6.25, 12.5, 18.75 and 25 at. % for the γNb_5_Si_3_. Higher Ti concentrations of 37.5 at. % and 50 at. % were considered in order to study the effect of the Ti concentration on the stability of the hexagonal silicide, and provide an estimation of the critical Ti concentration to form γNb_5_Si_3_. *Ab initio* technique has been used previously to study the effects of alloying on stability and mechanical properties of αNb_5_Si_3_ [20–22]. In the first-principles study by Chen et al. [21] they considered the effect of the substitution of Nb by Ti on the stability of Nb_5_Si_3_. Chen et al. studied only the substitution of one atom of Nb with Ti (i.e. alloying with 3.125 at. % Ti) on different atomic positions at 0 K.

**Figure 1.**
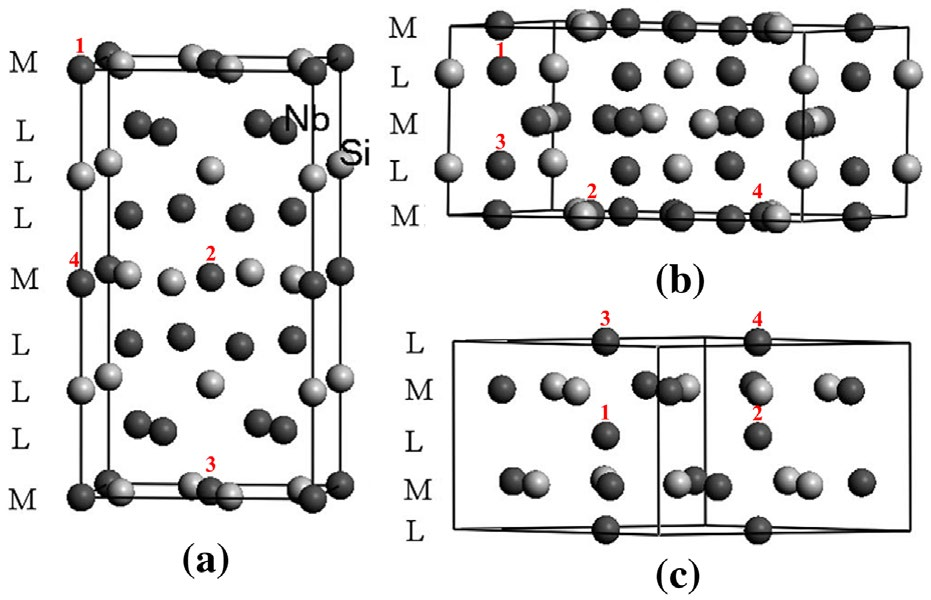
Sites of preference of Ti substituting Nb atoms in (a) alpha D8_l_, (b) beta D8_m_ and (c) gamma D8_8_ silicides. The numbers above each atom show the sequence of site occupation by the Ti atoms, reproduced from Chen et al. [21]. Reproduced with permission from American Physical Society.

Figure 1 shows the crystal structures of the 5–3 silicide polymorphs. Ti can substitute Nb in all three polymorphs and occupies the more closely packed Nb sites in αNb_5_Si_3_ and the less closely packed Nb sites in βNb_5_Si_3_ and γNb_5_Si_3_ [21,22]. In Figure 1, M and L, respectively, represent the more and the less closely packed sites. In the work presented in this paper, in order to investigate the order of the site occupancies of Ti atoms with increasing Ti concentration, separate geometry optimizations were made, and the enthalpies of formation at 0 Κ were computed (Table 1). In the case of γNb_5_Si_3_ the enthalpies of formation for different combinations of occupancies were found to be approximately equal. The enthalpies of the most stable structures are indicated by bold numbers in Table 1.

**Table 1.** Enthalpies of formation at 0 K (kJ/mol) for all combinations of site occupancies of Ti substituting Nb atoms in αNb_5_Si_3_ and βNb_5_Si_3_ for Ti addition from 1 to 4 atoms. See also Figure 1 for reference to atom positions. The bold values are the enthalpies of the most stable structures.

	αNb_5_Si_3_	βNb_5_Si_3_
1 Ti atom (Nb 1)	**−64.513**	**−60.771**
1 Ti atom (Nb 2)	−64.481	−60.770
1 Ti atom (Nb 3)	−64.456	−60.770
1 Ti atom (Nb 4)	−64.461	−60.770
2 Ti atoms (Nb 1, Nb 2)	**−66.125**	**−61.956**
2 Ti atoms (Nb 1, Nb 3)	−65.981	−61.284
2 Ti atoms (Nb 1, Nb 4)	−66.105	−61.287
3 Ti atoms (Nb 1, Nb 2, Nb 3)	**−67.533**	**−62.528**
3 Ti atoms (Nb 1, Nb 2, Nb 4)	−67.535	−62.527
4 Ti atoms (Nb 1, Nb 2, Nb 3, Nb 4)	**−68.884**	**−63.143**

Using the enthalpies of formation and equation 1 [21], the impurity formation energies 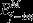
were calculated and are shown in Table 2.

**Table 2.** Enthalpies of formation and impurity formation energies at 0 K of the silicides of this study.

		Enthalpy of formation (kJ/mole)	Impurity formation energy (eV)
αNb_5_Si_3_	[16]	−62.841	
βNb_5_Si_3_	[16]	−59.654	
γNb_5_Si_3_	this work	−53.739	
γNb_5_Si_3_	[39]	−60.1	
αNb_19_Ti_1_Si_12_	this work	−64.513	−0.01733
αNb_18_Ti_2_Si_12_	this work	−66.125	−0.01671
αNb_17_Ti_3_Si_12_	this work	−67.533	−0.01459
αNb_16_Ti_4_Si_12_	this work	−68.884	−0.01400
αNb_8_Ti_12_Si_12_	this work	−69.306	
αNb_4_Ti_16_Si_12_	this work	−69.242	
βNb_19_Ti_1_Si_12_	this work	−60.771	−0.01158
βNb_18_Ti_2_Si_12_	this work	−61.956	−0.01228
βNb_17_Ti_3_Si_12_	this work	−62.528	−0.00593
βNb_16_Ti_4_Si_12_	this work	−63.143	−0.00637
βNb_8_Ti_12_Si_12_	this work	−67.057	
βNb_4_Ti_16_Si_12_	this work	−69.382	
γNb_9_Ti_1_Si_6_	this work	−57.036	−0.03417
γNb_8_Ti_2_Si_6_	this work	−60.385	−0.03471
γNb_7_Ti_3_Si_6_	this work	−62.077	−0.01754
γNb_6_Ti_4_Si_6_	this work	−63.929	−0.01920
γNb_8_Ti_12_Si_12_	this work	−66.607	
γNb_4_Ti_16_Si_12_	this work	−69.882	
Ti_5_Si_3_	this work	−72.888	
Ti_5_Si_3_	[40]	−74 ± 2	


(1)
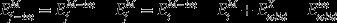



In equation 1 *M* and *X* denote the silicide and the substituted atom, respectively. The 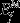 and 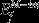 refer to the pure *M* (unalloyed) and impurity-doped (alloyed) *M* structures, 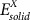 and 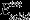 are the total energies of *X* and impurity atoms in their bulk states, respectively, and 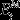 and 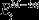 denote the total energies of the unit cell of *M* and impurity-doped *M* at their equilibrium state. Negative impurity formation energy means that the impurity-doped (alloyed) phase is more stable than the unalloyed phase, while the lower the impurity formation energy is, the more stable the doped (alloyed) phase. The Ti-doped structures exhibited negative impurity formation energies, which confirmed the study by Chen et al. [21], where the impurity formation energies for Ti in Nb_5_Si_3_ at T = 0 K were calculated. It can be seen in Table 2 that all the impurity formation energy values for all polymorphs were negative. The alloyed phase is more stable, the lower the impurity formation energy is. In all cases the impurity formation energy became more negative with each additional Ti atom, indicating increasing stability with increasing Ti substitution for all polymorphs.

The lattice constants and the volumes of the crystal structures of the 5–3 silicide polymorphs in the present study were calculated (Table 3). The a and c lattice parameters of the αNb_5_Si_3_ decreased and increased, respectively, as the Ti concentration increased. In the case of the βNb_5_Si_3_ and γNb_5_Si_3_ polymorphs both lattice parameters decreased as the Ti content increased. The lowest lattice parameters for the γNb_5_Si_3_ polymorph were for the case where all the Nb atoms are substituted by Ti atoms (i.e. for the Ti_5_Si_3_). The volume of all the 5–3 silicide polymorphs decreased as the Ti content increased, which is expected as Nb has a larger atomic radius than Ti [23,24].

**Table 3.** Lattice parameters and volumes of the studied intermetallic structures.

	Lattice parameter (Å)		Volume (Å^3^)
	a/b	c	
αNb_5_Si_3_ [16]	6.6281	11.7973	518.283
βNb_5_Si_3_ [16]	10.0686	5.0828	515.278
γNb_5_Si_3_	7.5706	5.2696	261.556
αNb_19_Ti_1_Si_12_	6.5854	11.9191	516.905
αNb_18_Ti_2_Si_12_	6.5704	11.9225	514.692
αNb_17_Ti_3_Si_12_	6.5565	11.9242	512.597
αNb_16_Ti_4_Si_12_	6.5427	11.9253	510.485
βNb_19_Ti_1_Si_12_	10.0687	5.061	513.08
βNb_18_Ti_2_Si_12_	10.0666	5.0468	511.428
βNb_17_Ti_3_Si_12_	10.0679	5.0223	509.074
βNb_16_Ti_4_Si_12_	10.0662	5.0062	507.271
γNb_9_Ti_1_Si_6_	7.5593	5.2353	259.082
γNb_8_Ti_2_Si_6_	7.5479	5.1977	256.447
γNb_7_Ti_3_Si_6_	7.535	5.1753	254.465
γNb_6_Ti_4_Si_6_	7.5207	5.1513	252.331
Ti_5_Si_3_	7.464	5.1387	247.926

**Figure 2.**
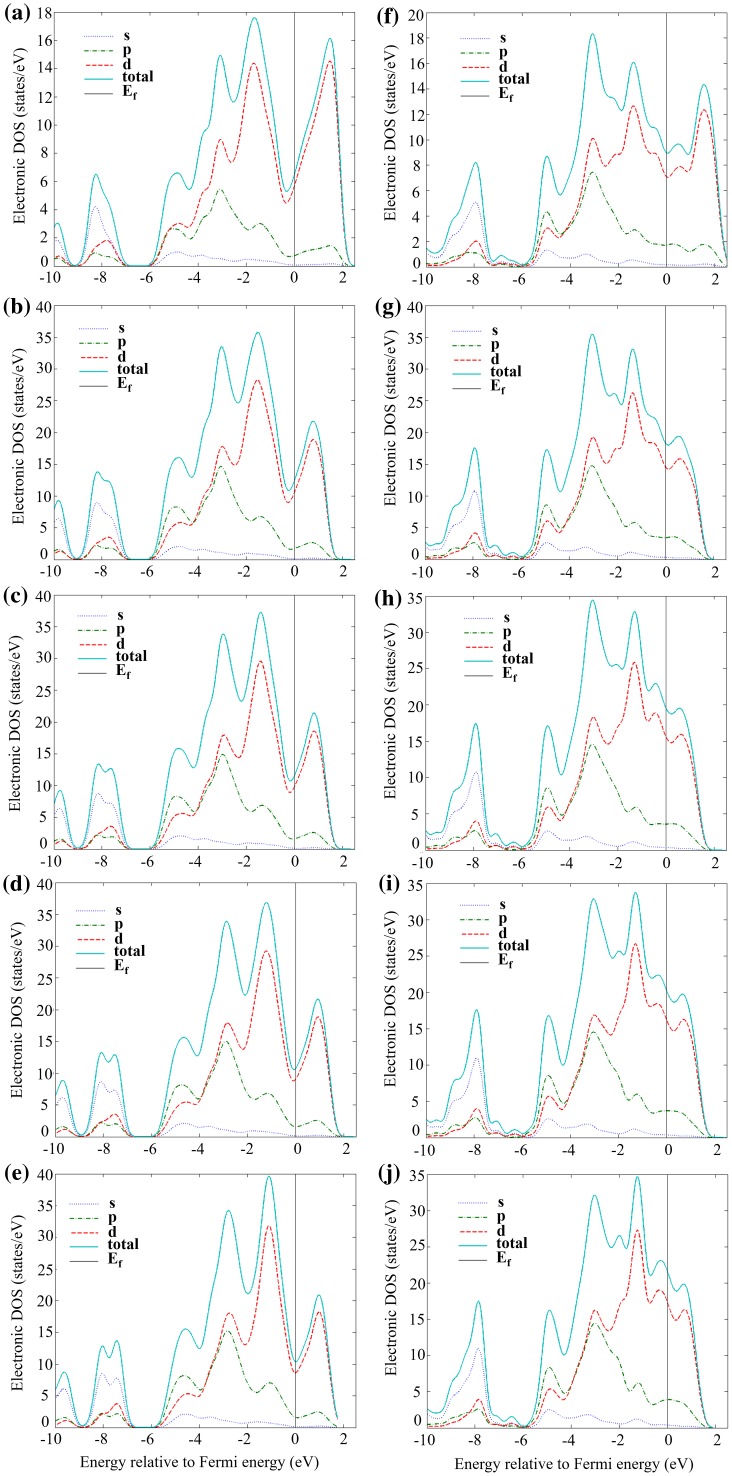
Partial and total density of states of (a) αNb_5_Si_3_, (b) αNb_19_TiSi_12_, (c) αNb_18_Ti_2_Si_12_, (d) αNb_17_Ti_3_Si_12_, (e) αNb_16_Ti_4_Si_12_, (f) βNb_5_Si_3_, (g) βNb_19_TiSi_12_, (h) βNb_18_Ti_2_Si_12_, (i) βNb_17_Ti_3_Si_12_ and (j) βNb_16_Ti_4_Si_12_ **.**

**Figure 3.**
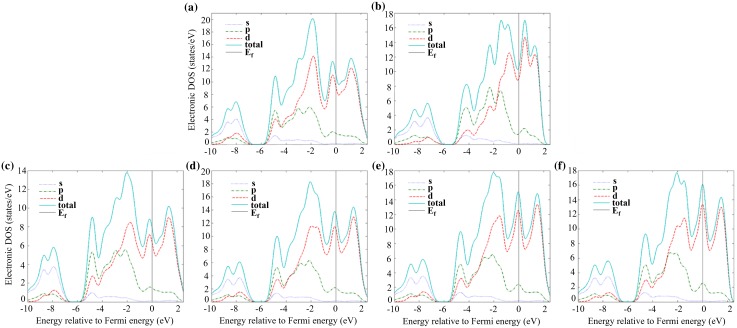
Partial and total density of states of (a) γNb_5_Si_3_, (b) Ti_5_Si_3_, (c) γNb_9_TiSi_6_, (d) γNb_8_Ti_2_Si_6_, (e) γNb_7_Ti_3_Si_6_, (f) γNb_6_Ti_4_Si_6_.

**Figure 4.**
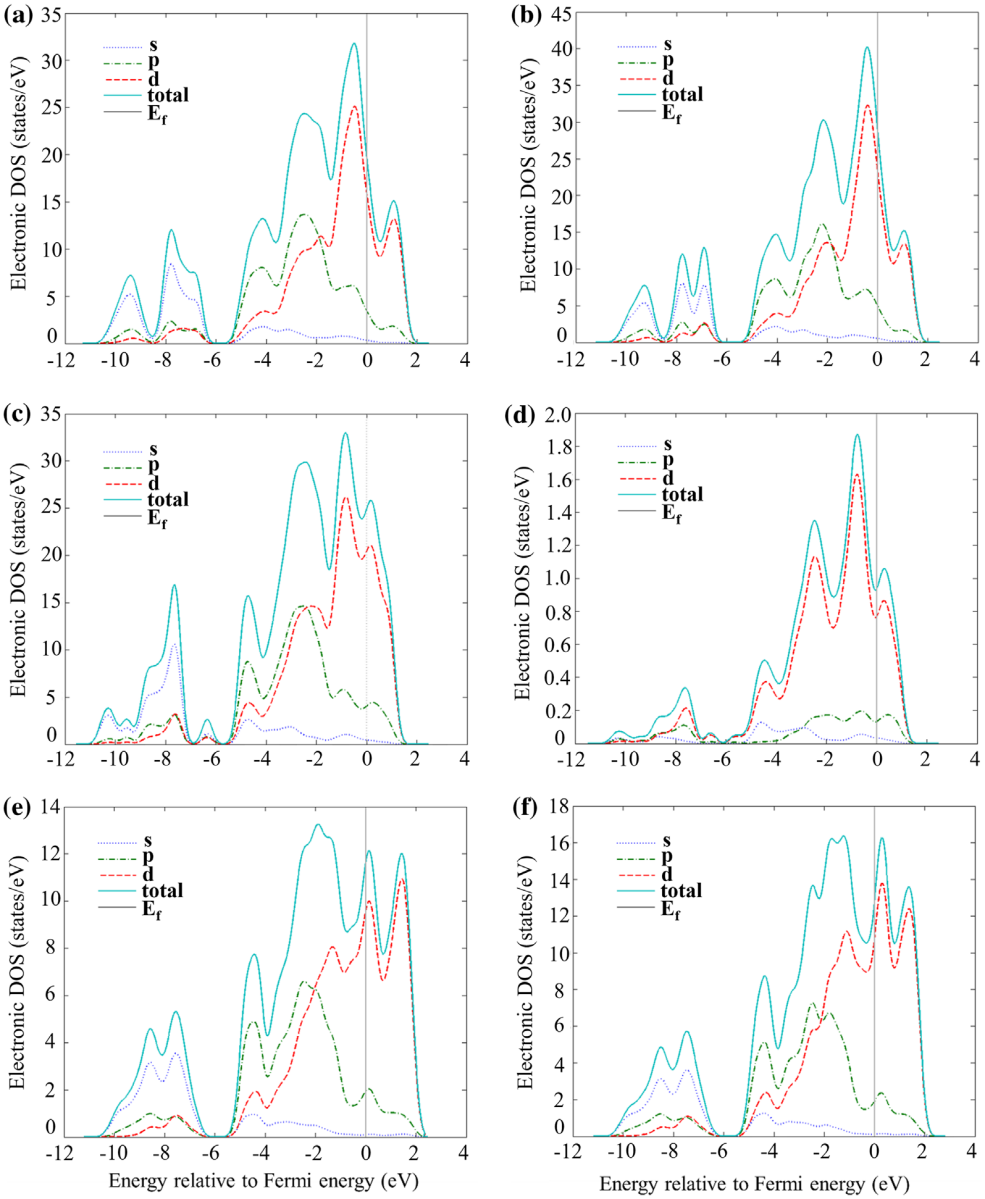
Partial and total density of states of (a) αNb_8_Ti_12_Si_12_ (37.5 at. % Ti), (b) αNb_4_Ti_16_Si_12_ (50 at. % Ti), (c) βNb_8_Ti_12_Si_12_ (37.5 at. % Ti), (d) βNb_4_Ti_16_Si_12_ (50 at. % Ti), (e) γNb_8_Ti_12_Si_12_ (37.5 at. % Ti), (f) γNb_4_Ti_16_Si_12_ (50 at. % Ti)**.**

The partial (PDOS) and total (TDOS) electronic densities of states are shown in Figures 2 to 4 for the α, β and γ 5–3 silicide structures. It can be seen that for all structures the main contribution to the TDOS was the PDOS of d electron states, followed by the p electron states, while the s electron states contribute the least to the TDOS of all structures.

The location of the Fermi level is indicative of phase stability. If the Fermi level is located in a deep valley of the TDOS, this indicates phase stability, whereas the opposite is the case if the Fermi level is located near peaks of the TDOS. It is clear that for the unalloyed compounds the valleys near the Fermi levels were deeper in αNb_5_Si_3_ (Figure 2(a)) than βNb_5_Si_3_ (Figure 2(f)), whereas for the γNb_5_Si_3_ (Figure 3(a)) the Fermi level is situated near one of the high peaks of the TDOS. This explains the gradual decrease of phase stability from α to β to γ 5–3 silicide.

The addition of Ti in the αNb_5_Si_3_ slightly moves the Fermi level to the bottom of the deepest valley (Figure 2(b)–(e)), making the silicide even more stable, while in the case of βNb_5_Si_3_ the Fermi level moves slightly towards one of the small peaks (Figure 2(g)–(j)) rendering the silicide somewhat less stable. This confirms that the difference between the formation enthalpies of the α and β phases is increased as the aforementioned phases are alloyed (doped) with Ti. In the case of γNb_5_Si_3_, the Ti addition also moves the Fermi level slightly closer to one of the peaks (Figure 3(c)–(f)).

The evolution of the TDOS as the Ti concentration in γNb_5_Si_3_ increases shows that the Fermi level would pass the large peak and move towards the valley below, as the Ti content increases above 37.5 at. % (Figure 4(e), (f)). On the other hand, it can be seen in Figure 4(a), (b) and (c), (d) that the Fermi level moves away from the respective pseudo-gaps for the α and β polymorphs. This, combined with the enthalpies of formation of the aforementioned phases (see Table 2), confirms that the hexagonal γNb_5_Si_3_ silicide becomes stable compared with the other two tetragonal silicides when the Ti concentration reaches 50 at. %. Li et al. [12] reported that in a cast Nb-25Si-40Ti (at. %) alloy, the βNb_5-x_(Ti)_x_Si_3_ was the primary phase, whereas in the cast Nb-30Si-45Ti (at. %), Nb-25Si-45Ti (at. %) and Nb-25Si-60Ti (at. %) alloys the γTi_5-x_(Nb)_x_Si_3_ was the primary phase formed during solidification. The microstructures of the alloys studied by Li et al. [12] were not at equilibrium, nevertheless their data suggest that the hexagonal 5–3 silicide becomes stable at high Ti concentrations in excess of 40–45 at. %, which is in agreement with the present study.

### 3.2. Elastic properties

The results of the calculations of the independent elastic constants (C_ij_), bulk moduli (B) from elastic constants according to the Voigt–Reuss–Hill (VRH) scheme and bulk moduli and first pressure derivatives of bulk moduli (B’) from the Birch–Murnaghan equation of state (B-M EOS) for all compounds and elements are shown in Table 4. The mechanical stability criteria [19] were met for all phases. The elastic constants for the pure elements were in agreement with the experimental data [25–27]. The property data for the un-doped αNb_5_Si_3_, βNb_5_Si_3_ and γNb_5_Si_3_ from the literature [16] are also given in Table 4. Compared with the VRH scheme, the values obtained by the B-M EOS tend to be larger. There is good agreement between the values from the two calculations. The bulk modulus tends to decrease with increasing Ti concentration in all 5–3 silicides. The calculated values of shear modulus (G) and Young’s modulus (E) are given in Table 5. For the αNb_5_Si_3_ and γNb_5_Si_3_ silicides the shear and Young’s moduli tend to increase with increasing Ti addition. In the case of βNb_5_Si_3_ the corresponding values decrease.

**Table 4.** Elastic constants (C_ij_) and bulk modulus (B) in GPa for elements and silicides of this study.

		VRH approximation							Birch–Murnaghan EOS	
		C_11_	C_12_	C_13_	C_33_	C_44_	C_66_	B	B	B’
Nb	this work	241	126.3			26.7		164.5	165.1	4
	[26]	253	133			31				
Si	this work	151.2	57.4			73.1		88.7	91.2	4
	[27]	166	64			79.6				
Ti	this work	149.6	97.5	79.7	186.1	33		110.9	118.4	4
	[25]	160	90	66	181	46.5				
αNb_5_Si_3_	[16]	362.2	103.9	118.1	312.6	121.9	109.9	190.6	204	6
βNb_5_Si_3_	[16]	367.2	117.2	109.6	306.1	88.1	128.7	189.6	197.9	5
γNb_5_Si_3_	this work	319.3	147.8	94.1	342.2	43.4	85.8	183.5	188.3	5
αNb_19_Ti_1_Si_12_	this work	374.1	94.1	115.6	321.7	120.3	109.9	191	194.4	5
αNb_18_Ti_2_Si_12_	this work	373.1	91.3	114.3	322.9	133.5	122.5	189.8	192.8	5
αNb_17_Ti_3_Si_12_	this work	370.4	88.5	112.9	322.4	134.1	124.4	187.9	190.5	5
αNb_16_Ti_4_Si_12_	this work	367.6	85.6	111.8	322.3	135.5	126	186.2	187.6	5
βNb_19_Ti_1_Si_12_	this work	362.1	119.1	108.1	308.3	83.3	128.8	188.5	196.3	5
βNb_18_Ti_2_Si_12_	this work	354.2	118.8	106.6	301.4	76	126.7	185.3	193.1	5
βNb_17_Ti_3_Si_12_	this work	347.1	118.3	106.3	298.3	70	126.4	184.2	192.3	5
βNb_16_Ti_4_Si_12_	this work	341	118.4	105.7	295.1	68.8	127.1	181.2	188.1	5
γNb_9_Ti_1_Si_6_	this work	314.3	142.2	89.8	332.3	49	86.1	178.1	181.3	5
γNb_8_Ti_2_Si_6_	this work	308.2	138	84.9	324.6	54.2	85.1	172.8	175.6	5
γNb_7_Ti_3_Si_6_	this work	299.3	134.3	81.2	314.5	59.3	82.5	167.2	171	5
γNb_6_Ti_4_Si_6_	this work	293.8	129.6	76.3	305.2	64	82.1	161.6	163.1	5
Ti_5_Si_3_	this work	273	113.5	54.5	259.7	86.5	79.8	138.1	140	5
Ti_5_Si_3_	[32] (calc.)	282.12	116.35	59.47	261.46	91.56	82.89	143.1		
Ti_5_Si_3_	[32] (exp.)	285	106	53	268	93	89.3			

**Table 5.** Calculated shear (G) and elastic (E) moduli in GPa, Poisson’s ratio (v), Cauchy pressures (C_12_-C_44_ for cubic, C_13_-C_44_ and C_12_-C_66_ for tetragonal and hexagonal) in GPa, G/B ratio and Debye temperature (Θ_D_) from elastic constants and phonon DOS for elements and silicides.

	G	E	v	C_12_-C_44_	C_13_-C_44_	C_12_-C_66_	G/B	Phonon DOS	Θ_D_ (K)	
	VRH								Elastic constants	Literature
Nb	36.5	101.9	0.396	99.6			0.228	277	268	
Exp. [28]	37.5	104.9	0.397							275
Exp. [29]										267
Calc. [30]	36.6									266
Si	61.2	149.2	0.216	−17.4			0.701	647	628	
Exp. [31]	64.1	155.8	0.215							645
Exp. [29]										646
Calc. [30]	58.2									608
Ti	32.7	89.3	0.366		19.5		0.295	369	346	
Exp. [31]										380
αNb_5_Si_3_ [16]	116.8	291	0.246		−3.8	−6	0.613	512	532	
βNb_5_Si_3_ [16]	106.4	268.9	0.263		21.5	−1.5	0.561	489	508	
γNb_5_Si_3_	71.2	188.5	0.324		50.7	62	0.388	401	420	
αNb_19_Ti_1_Si_12_	126.1	310.1	0.229		−4.7	−15.8	0.660	533	557	
αNb_18_Ti_2_Si_12_	127.3	312.1	0.226		−19.2	−31.2	0.671	541	565	
αNb_17_Ti_3_Si_12_	127.9	312.7	0.222		−21.2	−35.9	0.681	550	572	
αNb_16_Ti_4_Si_12_	128.7	313.8	0.219		−23.7	−40.4	0.691	569	580	
βNb_19_Ti_1_Si_12_	103.7	262.9	0.268		24.8	−9.7	0.550	496	507	
βNb_18_Ti_2_Si_12_	98.5	251	0.274		30.6	−7.9	0.532	488	500	
βNb_17_Ti_3_Si_12_	95.3	243.8	0.279		36.3	−8.1	0.517	480	497	
βNb_16_Ti_4_Si_12_	93.1	238.5	0.281		36.9	−8.7	0.514	477	496	
γNb_9_Ti_1_Si_6_	74.5	196	0.315		40.8	56.1	0.418	412	438	
γNb_8_Ti_2_Si_6_	77	201.2	0.306		30.7	52.9	0.446	428	453	
γNb_7_Ti_3_Si_6_	78.5	203.5	0.296		21.9	51.8	0.469	436	467	
γNb_6_Ti_4_Si_6_	80.5	207.1	0.286		12.3	47.5	0.498	451	483	
Ti_5_Si_3_	88.6	219	0.236		−32	33.7	0.642	579	598	
Calc. [32]	91.8	227	0.236							

The Cauchy pressures (C_12_-C_44_ for cubic and C_13_-C_44_ and C_12_-C_66_ for tetragonal and hexagonal structures), Pugh’s [33] index of ductility (ratio of shear modulus over bulk modulus (G/B)) and Poisson’s ratio (v) were calculated. The values of the aforementioned properties are given in Table 5. These parameters are often used as ‘predictors’ of the ductile or brittle behavior of intermetallics. For metallic bonding, a positive or negative value of Cauchy pressures means respectively a ductile or brittle material [34]. The other two conditions for brittle behavior are G/B > 0.57 and ν < 0.26. The results of the present study would suggest that the most ductile of the unalloyed silicides is the γNb_5_Si_3_, and the least ductile is the αNb_5_Si_3_. The αNb_5_Si_3_ and γNb_5_Si_3_ silicides become more brittle as the Ti content increases, whereas the βNb_5_Si_3_ becomes more ductile.

The elastic moduli for different Ti concentrations in 5–3 silicides are given in Table 5. Elastic moduli reflect the cohesion in a crystal structure. For αNb_5_Si_3_ and γNb_5_Si_3_ the elastic modulus increases with increasing Ti concentration, whereas for βNb_5_Si_3_ the elastic moduli decrease. This suggests that the addition of Ti strengthens atomic bonding in αNb_5_Si_3_ and γNb_5_Si_3_, and reduces bond strength in βNb_5_Si_3_.

### 3.3. Enthalpies of formation, transition temperatures and thermal expansion coefficients

The vibrational density of states (DOS) for the elements and silicides of this study were calculated. All the eigenfrequencies were found to be real, hence it was confirmed that the silicides are mechanically stable. After inserting the computed phonon DOS in the relevant formulae the vibrational contribution to free energies per atom (F^phon^(T)) was calculated for the D8_l_, D8_m_ and D8_8_ structures. Data for the pure elements were shown previously in Papadimitriou et al. [16]. The F^phonon^ for both αNb_5_Si_3_ and βNb_5_Si_3_ silicides decreased faster as the Ti addition increased, whereas for the γNb_5_Si_3_, the F^phonon^ decreased more slowly as the Ti addition increased.

**Figure 5.**
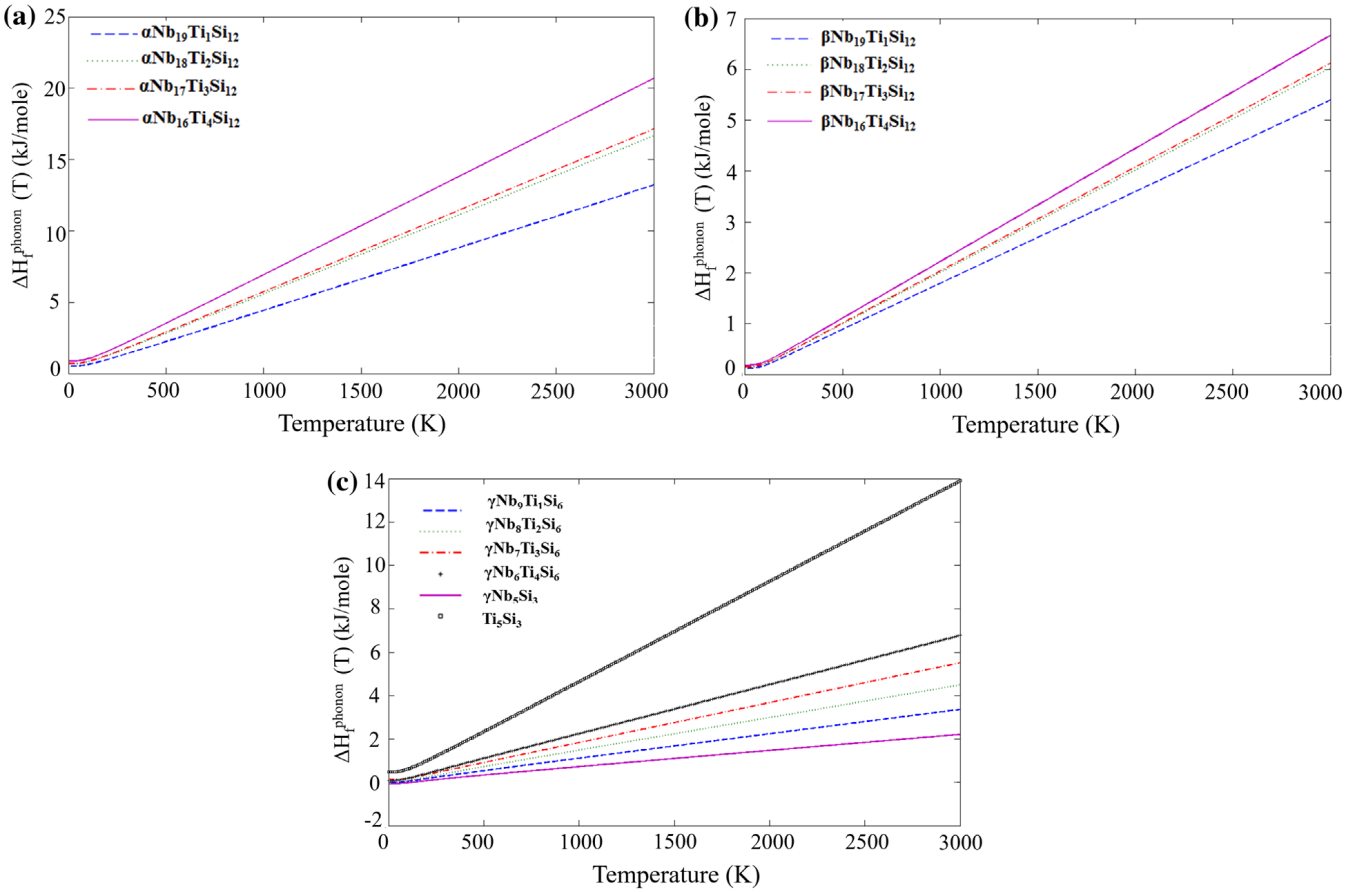
Vibrational contribution to the enthalpies of formation of the (a) D8_l_, (b) D8_m_, (c) D8_8_ silicides with Ti substitution**.**

After taking F^phonon^ into account, the phonon contribution to the enthalpy of formation (ΔH_f_^phon^ (T)) was evaluated for the D8_l_, D8_m_ and D8_8_ structures (Figure 5). For all the silicides the slope increased with increasing Ti addition. Comparison of the D8_l_ and D8_m_ structures shows that all values are significantly lower for the D8_m_. This shows that the temperature dependence of the phonon contribution favors the stability of the βNb_5_Si_3_ over αNb_5_Si_3_ with increasing temperature, which is expected from the binary phase diagram [3] and the experimental data for binary Nb-Si alloys. This trend is also followed by the Ti-alloyed phases, thus indicating that a Ti-alloyed βNb_5_Si_3_ should become more stable than the Ti-alloyed αNb_5_Si_3_ as the temperature increases.

**Figure 6.**
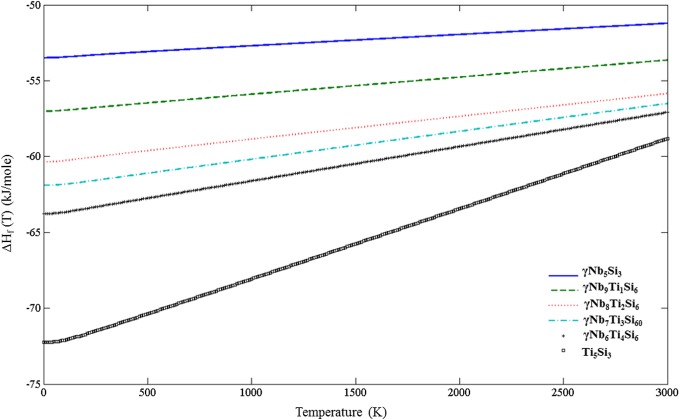
Enthalpies of formation of the D8_8_ silicides.

After acquiring the ΔH_f_ (T) for all unalloyed and alloyed phases, the phase equilibrium at finite temperatures was investigated. Figure 6 shows the enthalpy of formation of the γNb_5_Si_3_ for Ti content between 0 and 25 at. % and the enthalpy of formation of Ti_5_Si_3_. The slope of each curve increases as the Ti content increases from 0 at. % to fully Ti-alloyed 5–3 silicide, i.e. Ti_5_Si_3_. In all cases, over the whole temperature range, the Ti_5_Si_3_ has the lowest enthalpy of formation.

**Figure 7.**
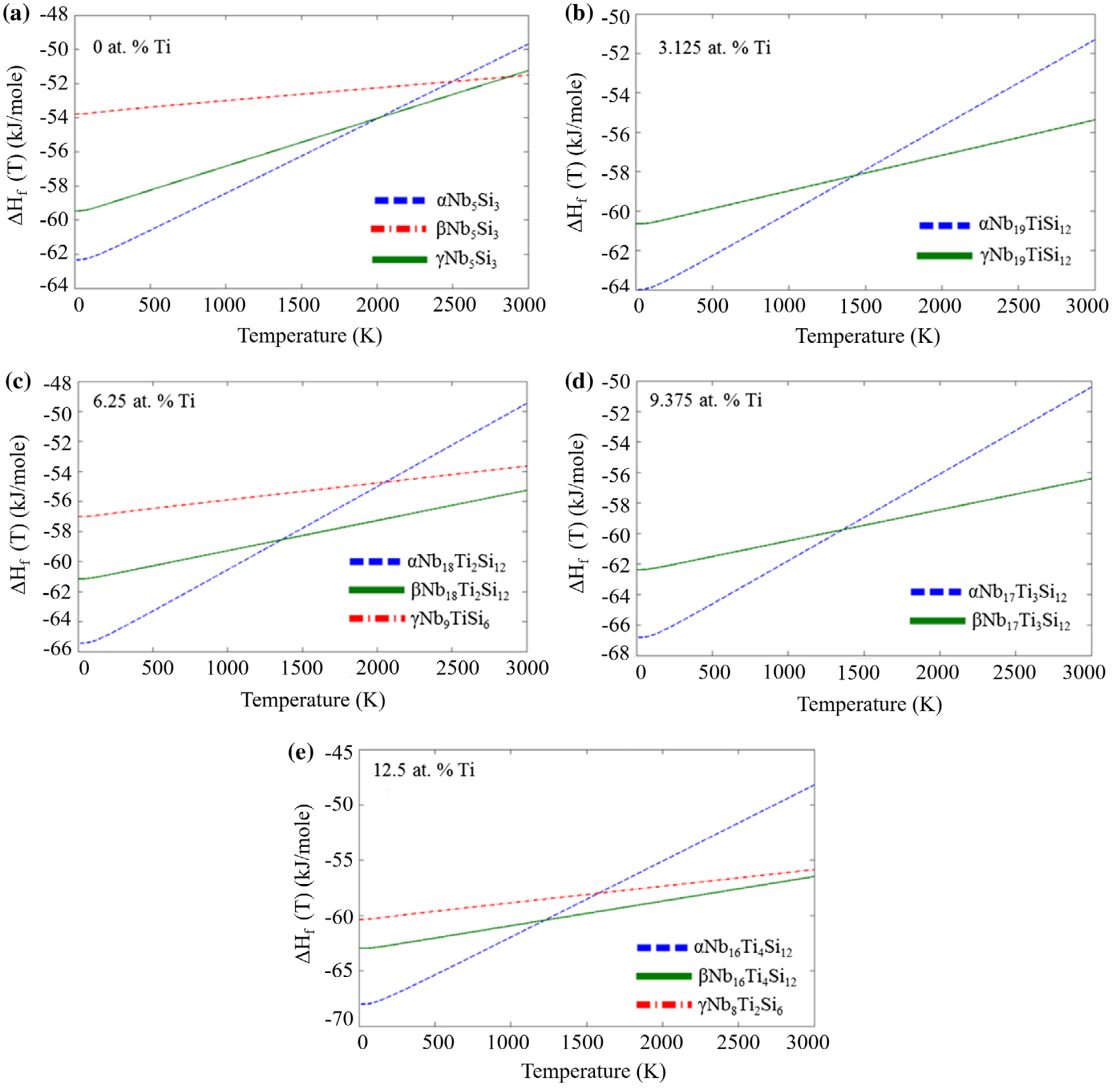
Enthalpies of formation of the alpha, beta and gamma silicides doped with 0 to 12.5 at. % Ti**.**

The enthalpy of formation against temperature of the D8_l_, D8_m_ and D8_8_ structures for up to 12.5 at. % Ti is shown in Figure 7. For all phases the enthalpy of formation increases with increasing temperature owing to the phonon contributions. Between 0 and 12.5 at. % Ti the γNb_5_Si_3_ is not expected to be stable. This is in agreement with experiments that show that this phase is metastable at low Ti contents. In Figure 7, for 0 at. % Ti, the γNb_5_Si_3_ curve does cross the βNb_5_Si_3_ curve; however, this occurs at a temperature above the melting temperature of both phases. Here the stable phase would be the liquid.

Comparing the unalloyed αNb_5_Si_3_ with the βNb_5_Si_3_ silicide, the former is stable up to 2085 K where its heat of formation curve crosses that of βNb_5_Si_3_, which becomes stable above this temperature (Figure 7(a)). This value is in good agreement with the transition temperature reported in the accepted Nb-Si binary phase diagram [3], as discussed in Papadimitriou et al. [16]. After adding Ti to the aforementioned structures this transition temperature decreases significantly to 1431 K for Nb_19_Ti_1_Si_12_, 1361 K for Nb_18_Ti_2_Si_12_, 1358 K for Nb_17_Ti_3_Si_12_ and finally to 1222 K for Nb_16_Ti_4_Si_12_ (Figure 7(a–e)). The contribution from the vibrational entropy is much greater for αNb_5_Si_3_ with increasing temperature, compared with βNb_5_Si_3_. Hence, the addition of Ti appears to have a larger effect on the phonon contribution of αNb_5_Si_3_, which drives the transition temperature lower. For the Nb-Si–Ti ternary system there are no experimental data with which to compare the calculated transition temperatures given above. In early experimental isothermal sections for similar temperatures [10,35] the prototype of Nb_5_Si_3_ was not stated. The error of finite temperature *ab initio* calculations can be large in some cases due to anharmonicity. Confidence in the above values is justified by the good agreement of the αNb_5_Si_3_ → βNb_5_Si_3_ transition temperature in the binary Nb-Si system with the literature.

Chen et al. [21] studied the stability of αNb_5_Si_3_ and βNb_5_Si_3_ when one Nb atom was substituted by a single Ti atom in its preferred site (e.g. the site with the lowest impurity energy) by comparing the differences in the calculated formation energies of the two silicides. They suggested that the larger the difference in formation energy, the higher the temperature of the phase transition. The difference between the enthalpy of formation at 0 K for unalloyed αNb_5_Si_3_ and βNb_5_Si_3_ and alloyed with 3.25 at. % Ti α and β Nb_5_Si_3_ (1 Nb atom replaced by Ti) increases with the Ti addition and is comparable with the results in Chen et al. [21]. Thus, based on the assumption of Chen et al., this would suggest that Ti will stabilize αNb_5_Si_3_ over βNb_5_Si_3_, and therefore the transition temperature would be expected to be pushed to higher values. Our results indicate the opposite trend, with Ti addition stabilizing βNb_5_Si_3_ and decreasing the transition temperature. For αNb_5_Si_3_ alloyed with Ti the temperature dependence of the phonon contribution to the heat of formation is much greater than that for βNb_5_Si_3_ alloyed with Ti, and therefore the slope of the ΔH_f_(T) curve for αNb_5_Si_3_ increases more dramatically with increasing temperature than for βNb_5_Si_3_. This indicates the importance of entropic contributions on phase stability that should be accounted for when considering the effect of alloying on transformation temperatures. In a thermodynamic assessment of the Nb-Ti–Si ternary system [11] the model used suggests that the stability of αNb_5_Si_3_ increases with increasing Ti content, and that αNb_5_Si_3_ alloyed with Ti becomes stable above the melting temperature of unalloyed αNb_5_Si_3_. The results of the present study suggest that a new assessment of the Nb-Ti–Si ternary system is needed.

The linear thermal expansion coefficients of the stable (tetragonal α and β) unalloyed Nb_5_Si_3_ and two Ti-alloyed silicides, namely the αNb_16_Ti_4_Si_12_ and βNb_16_Ti_4_Si_12_, are shown in Table 6. Also included in Table 6 are experimental values for Ti_5_Si_3_ [9]. There is good agreement between the calculated values and the available data in the literature. The CTE of all the silicides is anisotropic. The Ti_5_Si_3_ is the most anisotropic, whereas αNb_5_Si_3_ is the least. Alloying with 12.5 at. % Ti decreases the thermal expansion coefficients of both αNb_5_Si_3_ and βNb_5_Si_3_ silicides. However, the addition of Ti does not have a strong effect on the CTE anisotropy of both the α and β Nb_5_Si_3_.

**Table 6.** Linear thermal expansion coefficients (α_a_ and α_c_) for αNb_5_Si_3_, βNb_5_Si_3_, αNb_16_Ti_4_Si_12_ and βNb_16_Ti_4_Si_12_ at 298 K in 10^−6^/K.

Phase	α_a_	α_c_	α_a_/α_c_
αNb_5_Si_3_ (this work)	8.691	11.095	0.783
Experimental [36]	6.510	8.140	0.799
Experimental [9]	8.638	12.359	0.699
Experimental [37]	7.264	8.657	0.839
Theoretical [38]	9.210	10.336	0.891
βNb_5_Si_3_ (this work)	8.777	13.331	0.658
Theoretical [38]	8.328	17.211	0.484
αNb_16_Ti_4_Si_12_ (this work)	8.510	10.682	0.797
βNb_16_Ti_4_Si_12_ (this work)	6.709	10.980	0.611

### 3.4. Debye temperatures

The phonon DOS was used to calculate the Debye temperature, as described in Papadimitriou et al. [16]. The calculated values (Table 5) are in good agreement with those calculated using the elastic constants. For the elements the results from the calculations based on phonon DOS and the elastic constants are in good agreement with the literature. Regarding the silicides studied in this paper, the Debye temperatures that were calculated using the two methods are also in good agreement. For the αNb_5_Si_3_ and γNb_5_Si_3_ silicides the Debye temperature increases with increasing Ti content, but for the βNb_5_Si_3_ the opposite is the case, and the Debye temperature decreases slightly as more Nb atoms are substituted by Ti atoms.

Referring to the study by Chen et al. [30], according to which at the same temperature the number of the excited acoustic modes responsible for the stabilization of βNb_5_Si_3_ with respect to αNb_5_Si_3_ increases with the Ti content, it is the softer shear modulus of the Ti-alloyed βNb_5_Si_3_ compared with the Ti-alloyed αNb_5_Si_3_ that leads to the stability of this phase. For example, in Table 5 the shear moduli (G) of unalloyed α and β Nb_5_Si_3_, respectively, are 116.8 and 106.4 GPa. Alloying αNb_5_Si_3_ with Ti increases the shear modulus from 126.1 to 128.7 GPa when the Ti content increases from 1 to 4 atoms, whereas for βNb_5_Si_3_ the shear modulus decreases from 98.5 to 93.1 GPa when the Ti content increases from 1 to 4 atoms. Therefore, as the concentration of Ti is increased, the difference in the shear moduli values also increases, and this results in a decrease of the transition temperature.

## 4. Conclusions

First-principles calculations were carried out for the D8_l_, D8_m_ and D8_8_ polymorphs of Nb_5_Si_3_ alloyed with Ti, and the constituent elements. The volume of all structures contracted as the Ti addition increased. Elastic constants, bulk, shear and Young’s moduli, Poisson’s ratio and Debye temperature were calculated. These calculations showed that as the Ti content increased the bulk moduli of all silicides decreased, while the shear and elastic moduli increased for αNb_5_Si_3_ and γNb_5_Si_3_ and decreased for βNb_5_Si_3_. The Debye temperatures of αNb_5_Si_3_ and γNb_5_Si_3_ and βNb_5_Si_3_, respectively, increased and decreased as the Ti addition increased. The calculations suggested that the γNb_5_Si_3_ is the most ductile polymorph. The elastic properties of this silicide are reported in this paper. The alloying with Ti makes the αNb_5_Si_3_, and γNb_5_Si_3_ silicides less ductile and βNb_5_Si_3_ more ductile. The transition temperature between the α and β structures decreases as more Ti is added, and at about 50 at. % Ti content the hexagonal silicide becomes stable over its tetragonal polymorphs. The αNb_5_Si_3_ and βNb_5_Si_3_ exhibit anisotropy of their coefficients of thermal expansion, with the latter being more anisotropic that the former. Alloying the aforementioned compounds with 12.5 at. % Ti decreases their thermal expansion coefficients α_a_ and α_c_ without significantly changing the ratio α_a_/α_c_.

The results of this study indicate that the Ti-alloyed αNb_5_Si_3_ should be the desirable silicide in Nb-silicide based alloys, and that careful consideration must be given to the transition temperature between the two phases. The transition temperatures of the 5–3 silicides alloyed with Ti must be studied experimentally.

## Disclosure statement

No potential conflict of interest was reported by the authors.

## Funding

This work was supported by Engineering and Physical Sciences Research Council [grant number EP/M005607/01].

## References

[1] Balsone SJ, Bewlay BP, Jackson MR, et al Materials beyond superalloys-exploiting high-temperature composites In: Hemker KJ, Dimiduk DM, Clemens H, et al, editors. Structural intermetallics 2001. Warrendale: TMS; 2001 p. 99–108.

[2] Tsakiropoulos P., Beyond nickel based superalloys In: Blockley R, Shyy W, editors. Encyclopedia of aerospace engineering. John Wiley & Sons, Ltd; 2010.

[3] Schlesinger ME, Okamoto H, Gokhale AB, et al The Nb-Si (Niobium-Silicon) system. J Phase Equilib. 1993;14:502–509. 10.1007/BF02671971

[4] Chan KS Alloying effects on fracture mechanisms in Nb-based intermetallic in-situ composites. Mater Sci Eng: A. 2002;329-331:513–522. 10.1016/S0921-5093(01)01502-7

[5] Geng J, Tsakiropoulos P, Shao G Oxidation of Nb-Si-Cr-Al *in situ* composites with Mo, Ti and Hf additions. Mater Sci Eng A. 2006;441:26–38. 10.1016/j.msea.2006.08.093

[6] Begley RT Colombium alloy development at Westinghouse In: Dalder ENC, Grobstein T, Olsen CS, editors. Evolution of refractory metals and alloys. Warrendale, PA: TMS; 1994 P. 29–48.

[7] Vellios N, Tsakiropoulos P The role of Fe and Ti additions in the microstructure of Nb-18Si-5Sn silicide-based alloys. Intermetallics. 2007;15:1529–1537. 10.1016/j.intermet.2007.06.001

[8] Li ZF, Tsakiropoulos P Study of the effect of Ti and Ge in the microstructure of Nb-24Ti-18Si-5Ge *in situ* composite. Intermetallics. 2011;19:1291–1297. 10.1016/j.intermet.2011.04.010

[9] Zhang LT, Wu JS Thermal expansion and elastic moduli of the silicide based intermetallic alloys Ti_5_Si_3_(X) and Nb_5_Si_3_ . Scr Mater. 1997;38:307–313. 10.1016/S1359-6462(97)00496-X

[10] Liang H, Chang YA Thermodynamic modeling of the Nb-Ti-Si ternary system. Intermetallics. 1999;7:561–570. 10.1016/S0966-9795(98)00073-9

[11] Geng T, Li C, Bao J, et al Thermodynamic assessment of the Nb-Si-Ti system. Intermetallics. 2009;17:343–357. 10.1016/j.intermet.2008.11.011

[12] Li Y, Li CR, Du ZM, et al As-cast microstructures and solidification paths of the Nb-Si-Ti ternary alloys in Nb_5_Si_3_-Ti_5_Si_3_ region. Rare Metals. 2013;32:502–511. 10.1007/s12598-013-0143-9

[13] Jânio Gigolotti JC, Coelho GC, Nunes CA, et al Experimental evaluation of the Nb-Si-Ti system from as-cast alloys. Intermetallics. 2017;82(1):76–92. 10.1016/j.intermet.2016.04.006

[14] Bulanova M, Fartushna I Nb-Si-Ti, Landolt-Börnstein New Series IV/11E3. Berlin Heidelberg: Springer; 2010.

[15] Clark SJ, Segall MD, Pickard CJ, et al First principles methods using CASTEP, Z. Kristall. 2005;220:567–570.

[16] Papadimitriou I, Utton C, Scott A, et al Ab initio study of the intermetallics in Nb-Si binary system. Intermetallics. 2014;54:125–132. 10.1016/j.intermet.2014.05.020

[17] Monkhorst HJ, Pack JD Special points for Brillouin-zone integrations. Phys Rev B. 1976;13:5188–5192. 10.1103/PhysRevB.13.5188

[18] Montanari B, Harrison NM Lattice dynamics of TiO_2_ rutile: influence of gradient corrections in density functional calculations. Chem Phys Lett. 2002;364:528–534. 10.1016/S0009-2614(02)01401-X

[19] Born M, Huang K Dynamical theory of crystal lattices. Oxford: Oxford University Press; 1956.

[20] Shi S, Zhu L, Jia L, et al Ab-initio study of alloying effects on structure stability and mechanical properties of α-Nb5Si3. Comput Mater Sci. 2015;108:121–127. 10.1016/j.commatsci.2015.06.019

[21] Chen Y, Shang J-X, Zhang Y Bonding characteristics and site occupancies of alloying elements in different Nb_5_Si_3_ phases from first principles. Phys. Rev. B. 2007;76:184–204.

[22] Chen Y, Shang JX, Zhang Y Effects of alloying element Ti on alpha-Nb_5_Si_3_ and Nb_3_Al from first principles. J Phys Condens Matter. 2007;19:016215–16218. 10.1088/0953-8984/19/1/016215

[23] Slater JC Atomic radii in crystals. J Chem Phys. 1964;41:3199 10.1063/1.1725697

[24] Clementi E, Raimondi DL, Reinhardt WP Atomic screening constants from SCF functions. II. atoms with 37 to 86 electrons. J Chem Phys. 1967;47:1300–1307. 10.1063/1.1712084

[25] Tromans D Elastic anisotropy of hcp metal crystals and polycrystals. Int J Res Rev Appl Sci. 2011;6:462–483.

[26] Soderlind P, Eriksson O, Wills JM, et al Theory of elastic-constants of cubic transition-metals and alloys. Phys Rev B. 1993;48:5844–5851. 10.1103/PhysRevB.48.5844 10009117

[27] Simmons G, Wang H Single crystal elastic constants and calculated aggregate properties: a handbook. 2nd ed London: The M.I.T Press; 1971.

[28] Smithells CJ Metal references book. 5th ed London: Butterworth; 1976.

[29] Chu F, Lei M, Maloy SA, et al Elastic properties of C40 transition metal disilicides. Acta Mater. 1996;44:3035–3048. 10.1016/1359-6454(95)00442-4

[30] Chen Y, Hammerschmidt T, Pettifor DG, Shang J-X, Zhang Y Influence of vibrational entropy on structural stability of Nb–Si and Mo–Si systems at elevated temperatures. Acta Mater. 2009;57:2657–2664. 10.1016/j.actamat.2009.02.014

[31] Kittel C Introduction to solid state physics. 7th ed New York, NY: John Wiley & Sons; 1996.

[32] Chen X, Zeng M, Wang R, et al First-principles study of (Ti_5−x_Mg_x_)Si_3_ phases with the hexagonal D8_8_ structure: Elastic properties and electronic structure. Comput Mater Sci. 2012;54:287–292. 10.1016/j.commatsci.2011.10.042

[33] Pugh SF Relations between the elastic moduli and the plastic properties of polycrystalline pure metals. Philos Mag. 1954;45:823–843. 10.1080/14786440808520496

[34] Pettifor DG Theoretical predictions of structure and related properties of intermetallics. Mater Sci Technol. 1992;8:345–349. 10.1179/mst.1992.8.4.345

[35] Zhao J-C, Jackson MR, Peluso LA Mapping of the Nb-Ti-Si phase diagram using diffusion multiples. Mater Sci Eng. 2004;372:21–27. 10.1016/j.msea.2003.08.008

[36] Schneibel JH, Rawn CJ, Watkins TR, Payzant EA Thermal expansion anisotropy of ternary molybdenum silicides based on Mo_5_Si_3_ . Phys Rev B. 2002;65:8725–134115. 10.1103/PhysRevB.65.134112

[37] Rodrigues G, Nunes CA, Suzuki PA, Coelho GC Lattice parameters and thermal expansion of the T-2-phase of the Nb-Si-B system investigated by high-temperature X-ray diffraction. Intermetallics. 2004;12:181–188. 10.1016/j.intermet.2003.09.015

[38] Xu WW, Han JJ, Wang CP, et al Temperature-dependent mechanical properties of alpha-/beta-Nb_5_Si_3_ phases from first-principles calculations. Intermetallics. 2014;46:72–79. 10.1016/j.intermet.2013.10.027

[39] Parthé E, Nowotny H Strukturuntersuchungen an Siliziden Structural studies for silicides Monatsh Chem. 1955;86:385–396. 10.1007/BF00903622

[40] Meschel SV, Kleppa OJ Standard enthalpies of formation of some 3d transition metal silicides by high temperature direct synthesis calorimetry. J Alloys Compounds. 1998;267:128–135. 10.1016/S0925-8388(97)00528-8

